# *Auricularia auricula*’s Exopolysaccharide Mitigates DSS-Induced Colitis Through Dectin–1-Mediated Immunomodulation and Microbiota Remodeling

**DOI:** 10.3390/ph18081085

**Published:** 2025-07-22

**Authors:** Luísa Coutinho Coelho, Luísa Dan Favilla, Thais Bergmann de Castro, Maria Carolina B. Di Medeiros Leal, Christian Hoffmann, Anamélia Lorenzetti Bocca

**Affiliations:** 1 Laboratory of Applied Immunology, Department of Cellular Biology, Institute of Biological Sciences, University of Brasilia, Federal District, Brasilia 70910-000, Brazil; luisa98@gmail.com (L.C.C.); luisadanfavilla@gmail.com (L.D.F.); bergmann.castro@gmail.com (T.B.d.C.); 2 Medicine Department, University Center of Planalto Central Aparecido dos Santos, Federal District, Brasilia 72445-020, Brazil; caroldimedeiros@gmail.com; 3 Department of Food Science and Experimental Nutrition, School of Pharmaceutical Sciences, University of São Paulo, São Paulo 05508-000, São Paulo, Brazil; c.hoffmann@usp.br; 4 Bi-Institutional Translational Medicine Platform, Oswaldo Cruz Foundation (Fiocruz), Ribeirão Preto 14049900, São Paulo, Brazil; 5 National Institute of Science and Technology in Human Pathogenic Fungi, Ribeirão Preto 14049900, São Paulo, Brazil

**Keywords:** ulcerative colitis, prebiotics, mushroom polysaccharide, microbiome

## Abstract

**Background/Objectives:** Ulcerative colitis (UC) is characterized by the interplay between immune responses and dysbiosis in disease development. Aiming to provide additional insights into disease development and potential treatment strategies, the present study investigates the local effect of oral treatment with polysaccharides obtained from *Auricularia auricula*’s submerged culture in an experimental model of DSS-induced colitis and its impact on lesion resolution. **Methods**: The structure and monosaccharide composition of *Auricularia* polysaccharides were characterized through Nuclear Magnetic Resonance (NMR). To evaluate the effect of this polysaccharide on the murine model, wild-type and Dectin-1 knockout mice were treated or not with the exopolysaccharide (EPS) while under DSS consumption. During the experimental period, feces samples were collected to evaluate microbial shifts during disease development, and, finally, the colonic tissue was analyzed to assess the inflammatory process and cytokine production. **Results**: The EPS composition showed a polymeric mixture of glucans and fucogalactomannans. The treatment of the wild-type DSS-induced colitis group improved the inflammatory response by increasing gut–homeostatic cytokines, such as interleukin-10 (IL-10) and tumor necrosis factor-alpha (TNF-α). The Dectin-1 KO mice group did not show the same enhancement after EPS treatment. The microbiome analysis revealed a difference in the genotype, and the treatment modified the DSS microbiome modulation, with nine and four ASVs in WT and Dectin-1 KO mice, respectively. **Conclusions**: The EPS treatment demonstrated therapeutic potential in treating inflammatory intestinal diseases by modulating cytokine secretion and microbiota composition, which is dependent on the Dectin-1 receptor’s carbohydrate recognition.

## 1. Introduction

Dysbiosis, characterized by reduced microbial diversity and shifts in bacterial populations, has been consistently documented in patients with ulcerative colitis (UC) and in murine models [1]. This condition, marked by an imbalance in intestinal microbiota composition, can be precipitated by various factors, including dietary changes, environmental exposure, and antibiotic use. This imbalance in the microbiota is widely associated with an increased susceptibility to inflammatory and metabolic pathologies, such as obesity, cardiometabolic diseases, cancer, and inflammatory bowel diseases (IBD), with ulcerative colitis being particularly noteworthy [2,3,4,5].

Recent studies have demonstrated that dysbiosis can exacerbate intestinal inflammation, mediated by effector T cells and pro-inflammatory cytokines, leading to aggravated clinical symptoms [6,7]. Particularly in UC, the activation of antigen-presenting cells, such as dendritic cells and macrophages, is closely linked to the exacerbated immune response observed in individuals with the condition. This phenomenon is characterized by the increased production of interferon-gamma (IFN-γ) and tumor necrosis factor-alpha (TNF-α), as well as the activation of the Th17 response, resulting in a cycle of chronic inflammation [4,8,9,10].

The role of pattern recognition receptor Dectin-1 in UC is crucial in the immune response against fungi, but it remains controversial in the literature. While some studies indicate that the deficiency of this receptor in mouse models of DSS-induced colitis decreases disease susceptibility through T-regulatory cell expansion [11], others suggest that it is associated with disease deterioration by the presence of fungal species in the gut [12,13] or bacteria [14]. The Dectin-1-Card9 axis is essential to controlling gut inflammation and to DSS-induced colitis development [13,15]. The administration of antifungals may also aggravate colitis and alter the microbiome [13,16].

Depending on the carbohydrate composition, Dectin-1 ligands can modulate tissue inflammation and regulate the production of inflammatory cytokines. Polysaccharides have gained prominence in exploring possible therapeutic interventions due to their immunomodulatory properties, especially those capable of promoting a highly tolerogenic profile, with the production of interleukin (IL)-10 controlling the inflammatory response. They can be a target for new therapeutic approaches in DSS-induced colitis [1,17,18,19].

The complex interaction of polysaccharides and gut microbiota may also provide substrates for gut microbiota, leading to the production of short-chain fatty acids (SCFAs), such as acetate, propionate, and butyrate, which play significant roles in modulating host health [20,21]. These metabolites impact local gastrointestinal immunity and systemic immune responses, underscoring their pleiotropic effects across various body systems [22,23]. Additionally, the fermentation of these carbohydrates by gut bacteria promotes advantageous shifts in gut microbiota, which plays a vital role in modulating inflammatory responses and supports metabolic functions essential for maintaining gut homeostasis [24,25,26,27]. As dysbiosis often results from dietary changes, environmental stressors, or antibiotic use, which may exacerbate inflammatory conditions and metabolic disorders [28,29], polysaccharides can help ameliorate such issues by restoring microbial balance and enhancing the production of beneficial SCFAs, thereby promoting barrier integrity and reducing inflammation [30,31,32]. Restoration of microbiota through polysaccharide treatments is a therapeutic axis in managing UC, promoting beneficial microbiota that suppress pro-inflammatory signals and enhance epithelial barrier functions. In this context, the present study investigates the effect of treatment with exopolysaccharides derived from *Auricularia auricula*’s submerged culture in an experimental DSS-induced colitis model, aiming to provide additional insights into potential management strategies.

## 2. Results and Discussion

### 2.1. NMR Analysis of Auricularia auricula Polysaccharides

**Figure 1:**
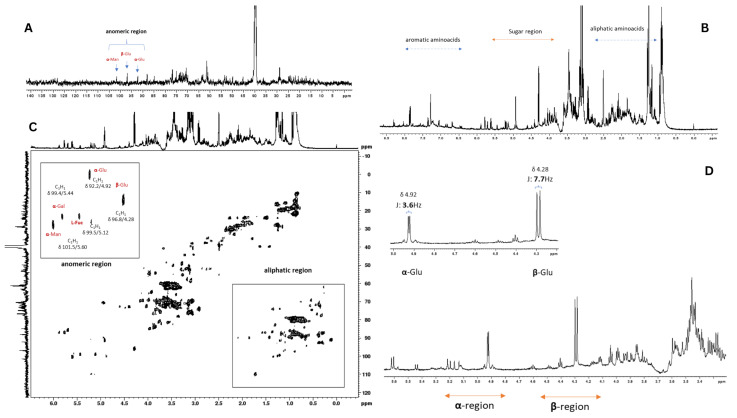
(**A**) ^13^C NMR spectrum of polysaccharides from *Auricularia auricula* (500 MHz, DMSO at 70 °C). (**B**) ^1^ H NMR spectrum (500 MHz, DMSO at 70 °C) (**D**), with an amplified insert of the anomeric region. (**C**) HSQC_edit_ spectra, with amplified inserts of aromatic and aliphatic region (500 MHz, DMSO at 70 °C).

To corroborate the results with previously collected data, the fine structure of *Auricularia* polysaccharides from the insoluble fraction was analyzed using 1D and 2D NMR (Figure 1). The chemical shift values are listed in Supplementary Table S1. The determination of typical residues combined results from methylation and monosaccharide analyses, as previously reported in the literature [33].

Previous monosaccharide composition analyses demonstrated that the polysaccharide material obtained by simple precipitation and filtration was heterogeneous, consisting primarily of glucose (33.7%), mannose (27.3%), galactose (15.4%), and fucose (20.1%). The NMR analyses (Figure 1A) confirmed this monosaccharide composition. They revealed the presence of a polymeric mixture, as evidenced by the spectral profile in which distinct intensity patterns were observed for glucose signals compared to those of other monosaccharides (Figure 1B,C).

This diversified distribution of constituent monosaccharides is characteristic of polymeric mixtures where different types of polysaccharides contribute their specific monosaccharide units to the global compositional profile. As defined by Cheng et al. (2017), many copolysaccharides are compositionally heterogeneous, containing variations in monosaccharide composition and sequence distributions, where the composition determined by spectroscopic methods provides average values of individual components [34]. The observed spectral patterns indicate the coexistence of homopolymers characteristic of glucans and heteropolymers consistent with fucogalactomannans. Different polysaccharides present distinct chemical environments for their monosaccharide constituents, resulting in variations in the relative intensities of NMR signals, which constitutes direct evidence of the mixed polymeric nature of the material [35].

The glucans in the material were configured as alpha and beta isomers, whose presence is ubiquitous in fungi of this nature (Figure 1D). The identification of α and β isomers in *Auricularia auricula* glucans was based on specific spectral evidence observed in the anomeric region of the ^1^H NMR spectrum (Figure 1B,D).

The identification of α and β isomers in *A. auricula* glucans was based on specific spectral evidence observed in the anomeric region of the ^1^H NMR spectrum (Figure 1B,D). Diagnostic spectral parameters were applied for this identification. In the amplified anomeric region (Figure 1D), anomeric H-1 protons of different configurations can be observed. This spectral region (typically 4.3–5.9 ppm) is diagnostic for anomer identification [36]. The spectral data show distinct signals in the anomeric region that are consistent with the presence of both anomers:

H-1 protons of β-glucans resonate at a higher field (approximately 4.5–4.7 ppm).

This differentiation is based on the characteristic behavior of α-glycoside protons, which typically exhibit chemical shifts at lower fields than the corresponding β-glycosides [4]. Additionally, HSQC edit spectra (Figure 1C) provide ^1^H-^13^C correlation that confirms the assignment of anomeric signals. The anomeric carbons C-1 of α and β-glucans present characteristic ^13^C chemical shifts:

C-1 β-glucans: approximately 97–99 ppm.

The presence of both α and β anomers in fungal glucans is consistent with the known structure of *Auricularia* polysaccharides. Previous studies have demonstrated that glucans from fungi of this species typically contain α-(1→3) and β-(1→3) linkages, as well as α-(1→6) and β-(1→6) branches, resulting in the coexistence of different anomeric configurations [33].

### 2.2. A. auricula Polysaccharides’ Activity in the Murine Colitis Model

**Figure 2:**
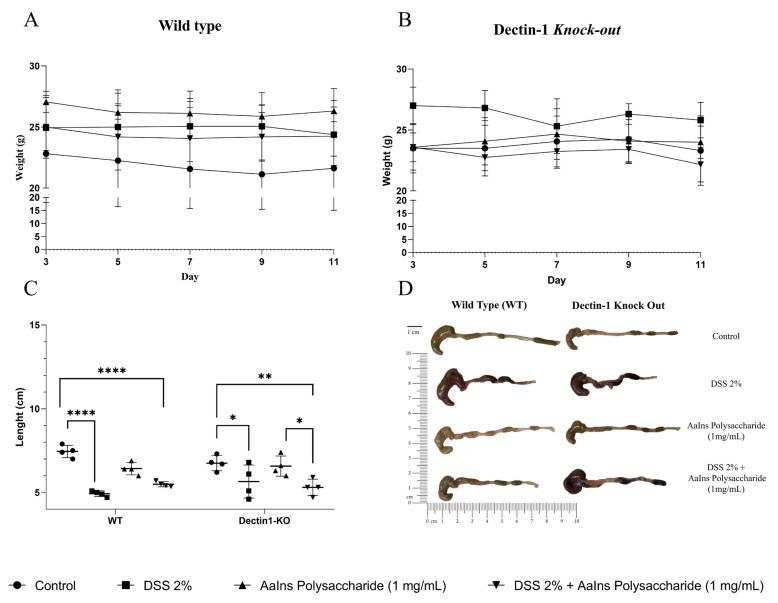
Effect of *A. auricula* polysaccharide on the mice’s body weight (g) and colon length (cm) according to the treatment (●) not treated, (◼) DSS 2%-induced-colites, (▲) treated with EPS, and (▼) treated with EPS and DSS 2%-induced-colites. (**A**) body weight curve of c57BL6/J wild-type mice through the experimental period; (**B**) body weight curve of c57BL6/J and Dectin-1 KO mice throughout the experimental period; (**C**) Graphical representation of colon length measured with ImageJ 1.53t. Mixed-effects analysis, where *p* < 0.05 compared to the control group; (**D**) Photograph showing the mice’s intestinal length. * *p* < 0.05; ** *p* < 0.005; and **** *p* < 0.0001; and compared to the group treated with EPS, one-way ANOVA and Tukey’s test.

The DSS-induced colitis model evaluated the activity of polysaccharides obtained from *A. auricula* in wild-type (WT) and Dectin-1 Knockout (KO) mice. The mice were treated with exopolysaccharide by gavage, and after three days, some received 2% DSS and continued to be treated throughout the experimental period. At the end of DSS treatment, the animals were euthanized for analysis. Our previous data showed that gut modulation by mushroom exopolysaccharide initiated at 3 days post-treatment; therefore, we maintained the treatment schedule. Although there were no significant changes in the mice’s body weight during the treatment (Figure 2A), we can observe in the comparative size graph (Figure 2C) that, as expected, the WT control group for colitis development showed a reduction in intestinal length following ingestion of the DSS 2% solution. The macroscopic recovery of colon length in the groups that received treatment with the exopolysaccharide or not is shown in Figure 2D. The shortening of the colon caused by inflammation is one of the clinical marker signs of colitis, as is the inflammatory reaction, which is observed in the histopathological analysis, with epithelial damage, Goblet cell destruction, and increase in the inflammatory cell infiltration when compared with the control mice (Supplementary Figures S1A,B and S2A,B). The treatment with exopolysaccharide did not modify the colon structure (Supplementary Figures S1C and S2C). The treatment before DSS-induced colitis improves the histopathological aspects, with reduced inflammatory cell infiltrate and preserved tissue structure in WT mice (Supplementary Figure S1D). However, this was not observed in Dectin-1 KO mice (Supplementary Figure S2D). The Dectin-1 KO mice exhibited a reduction in colon length in groups that received DSS, corroborating the histopathological findings. Although treating WT mice with the exopolysaccharide did not restore the colon length, the histopathological aspects showed an improvement in the DSS-induced tissue inflammation.

Despite the murine model’s evolution to spontaneous healing, DSS-induced colitis is a widely used experimental model of colitis. The inflammation triggered by DSS is acute, intense, and often complex to reverse, characterized by severe epithelial damage that disrupts the tight junctions and leads to barrier breakdown, accompanied by massive infiltration of immune cells and cytokine production [37]. Our results showed that the exopolysaccharide oral treatment can reverse part of this intense tissue inflammation in a Dectin-1 receptor-dependent manner.

The exopolysaccharides derived from *A. auricularia*’s submerged culture interact with macrophages’ Dectin-1 receptor, but not with TLR-2, TLR-4, Dectin-2, and Dectin-3 receptors [33], and the lack of this receptor in KO mice may reflect the absence of treatment response. The significant results observed here are Dectin-1 receptor-dependent. However, the role of Dectin-1 in DSS-induced colitis is not a consensus [11,12,13,14,15]. Its presence is consistently associated with an upregulation of inflammation, particularly when the fungal burden is elevated. Mice lacking Dectin-1 show altered susceptibility to colitis, depending on fungal colonization—sometimes less inflammation due to reduced cytokine signaling, but sometimes more due to poor fungal control [11,12,13].

### 2.3. Cytokine Production

**Figure 3:**
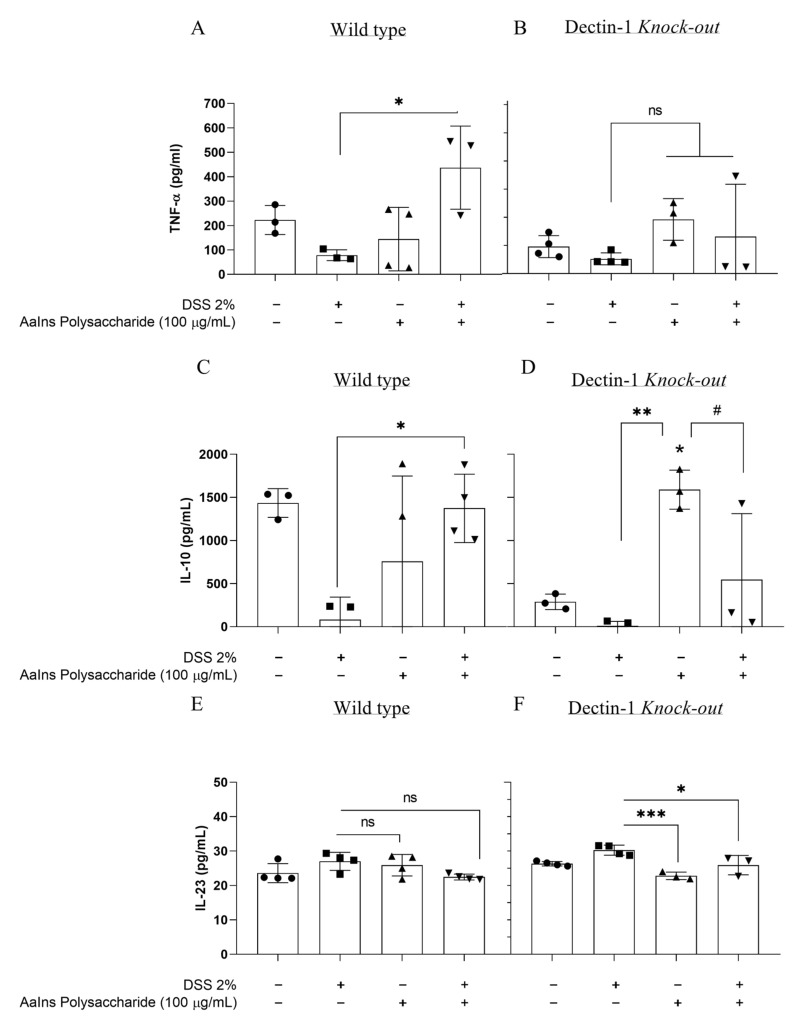
Quantifying cytokines measured in the colon of wild-type and Dectin-1 KO mice, given DSS 2% and treated/or not with *Auricularia* exopolysaccharide, according to the treatment (●) not treated, (◼) DSS 2%-induced-colites, (▲) treated with EPS, and (▼) treated with EPS and DSS 2%-induced-colites. (**A**,**B**) TNFα, (**C**,**D**) IL-10, (**E**,**F**) IL-23. Data are expressed as mean ± SD, compared to the DSS 2% group, where * *p* < 0.05; ** *p* < 0.005; and *** *p* < 0.001; and compared to the group treated with EPS, where # *p* < 0.05, one-way ANOVA and Tukey’s test.

**Figure 4:**
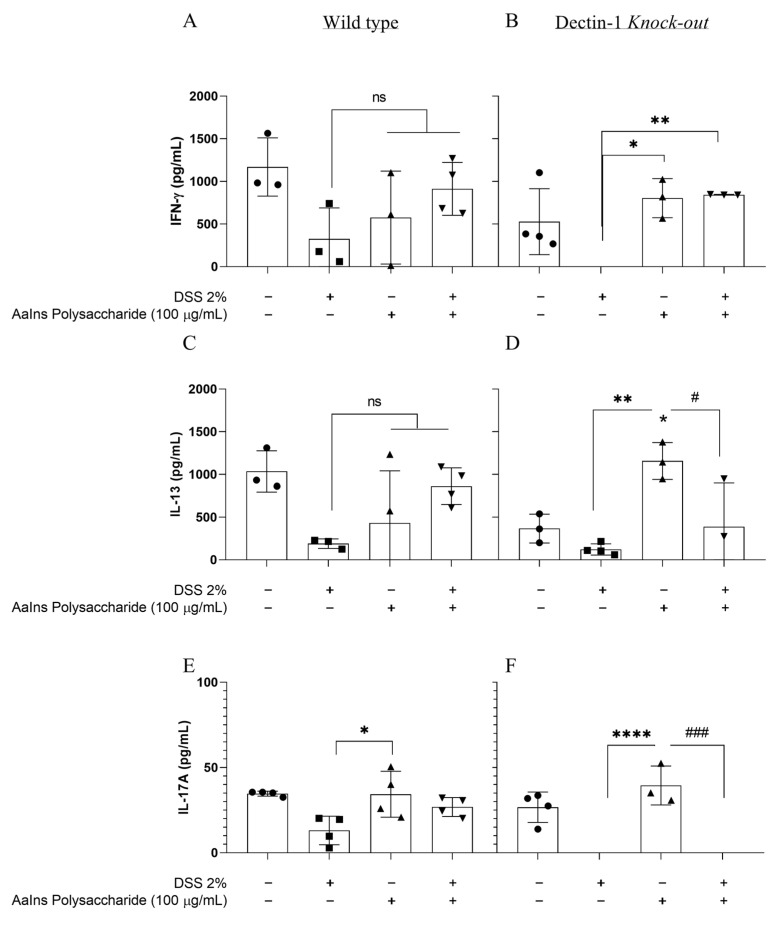
Quantifying cytokines measured in the colon of wild-type and Dectin-1 KO mice, given DSS 2% and treated/or not with *Auricularia* exopolysaccharide, according to the treatment (●) not treated, (◼) DSS 2%-induced-colites, (▲) treated with EPS, and (▼) treated with EPS and DSS 2%-induced-colites. (**A**,**B**) IFN-ɣ, (**C**,**D**) IL-13, (**E**,**F**) IL-17A. Data are expressed as mean ± SD, compared to the DSS 2% group, where * *p* < 0.05; ** *p* < 0.005; and **** *p* < 0.0001; and compared to the group treated with EPS, where # *p* < 0.05, and ### *p* < 0.001, one-way ANOVA and Tukey’s test.

The mushroom’s polysaccharides can modulate the host immune response, increasing the release of cytokines. To evaluate whether the treatment could intervene with inflammation induced by the colitis model, we profiled the cytokines TNF-α, IL-10, IL-23, IL-17A, IL-13, and IFN-γ in the intestinal macerate of the mice. We can observe that for WT mice with DSS-induced colitis, treatment with *A. auricula*’s polysaccharide enhanced the production of TNF-α in the colon (Figure 3A) and the IL-10 secretion (Figure 3C) without changes in IL-23 secretion (Figure 3E). There was no difference in IL-13, IL-17, and IFN-γ levels in this group (Figure 4A,C,E). In the gut, TNF-α plays a dual role, regulating tissue barrier functions by controlling the apoptosis of intestinal epithelial cells (IECs), the expression of tight junction proteins, and mucus secretion [38]. The strict balance between cell death and proliferation in the gut mucosa can mediate cell survival by activating NFκB signaling or triggering cell death, particularly caspase-dependent apoptosis, as well as caspase-independent programmed necrosis. Therefore, TNFα exhibits a range of beneficial functions in the intestine [39]. Nonetheless, it is associated with inflammatory bowel disease (IBD) by inducing a soluble natural antagonist of IL-22 (IL-22Ra2; IL-22BP) in the colon, thereby abrogating the IL-22/STAT3-mediated mucosal repair during colitis [40]. Moreover, T cells stimulated by TNF are associated with chronic colonic inflammation. In contrast, TNF produced by epithelial cells (IECs) induces IL-22BP expression in colonic dendritic cells (DCs) and dampens IL-22-driven restitution of colonic epithelial functions. In an inflammatory milieu, TNF-α increases cell infiltration in the gut.

Another cytokine important for maintaining gut homeostasis is IL-10, which plays a crucial role in regulating intestinal inflammation [38,41]. IL-10 was first reported as an inhibitory cytokine, but is now a well-studied cytokine. One important function is its anti-inflammatory effects, which involve suppressing the secretion of many pro-inflammatory molecules, inducing the secretion of anti-inflammatory molecules, and modulating CD4+ T-cell activation [42]. In the intestine, the sources and targets of IL-10 include leukocytes and epithelial cells. The absence of IL-10 induces spontaneous colitis early in life, associated with IL-22 but not IL-17 production in the development of inflammatory disease. It is proposed that IL-10 and IL-22 are associated in a functional network that counterbalances antimicrobial and tissue repair, as well as anti-inflammatory responses, to maintain immune microbial homeostasis in the gut [43]. The microbiota also influences the secretion of IL-10 through its metabolites, especially short-chain fatty acids (SCFAs). These metabolites upregulate the transcription factor B lymphocyte-induced maturation protein 1 (BLIMP-1) and, consequently, IL-10 release, thereby maintaining intestinal homeostasis [44]. Our results showed that EPS increased IL-10 and TNF-α production in the group that developed colitis and was treated with EPS. Based on the correlation with the production of these cytokines and histopathological analyses, we can conclude that EPS stimulated the homeostasis role of both cytokines and consequently decreased IL-22 production in the tissue.

The comparisons of DSS-induced colitis in Dectin-1 KO mice treated with or without EPS did not reveal differences in most cytokine levels. Still, with increased levels of IFN-γ (Figure 4F), the Dectin-1 KO mice showed increased levels of IL-10 (Figure 3D), IL-13 (Figure 4D), IFN-γ (Figure 4A), and IL-17 (Figure 4F) when only treated with EPS, revealing that the gut cells can respond to EPS stimulation through receptors other than Dectin-1.

The polysaccharides are composed mainly of glucans, which are recognized by host immune cells through Dectin-1, TLR-2, and Complement receptor 3 (CR3), and of galactomannans, which are identified by Dectin-2 [45]. Although the recognition of glucans is mainly attributed to Dectin-1, which promotes maximal induction of gene expression, it is worth noting that in the absence of this receptor, the polysaccharide may be stimulating the production of cytokines through other signaling pathways, as we observed in the Dectin-1 KO mice only treated with EPS (Figure 3B,D,F) [46,47].

Non-digestible carbohydrates, derived from dietary fibers such as polysaccharides, are metabolized by the intestinal microbiota and serve as substrates for bacterial fermentation [21]. It has been shown that not only does the fiber quantity of the diet impact SCFA production, but also that specific fiber types may be preferred by different species of bacteria in the gut, which have distinct fermentation patterns and produce differing SCFA quantities and, thus, provide alternative modulations in host metabolism, as described for IL-10 release [42,48,49]. The plasticity of the gut microbiota supports the associated biological outputs, with the immunity microbiota crosstalk being one of the most critical factors in this reshaping [50].

### 2.4. Beta Diversity Results

**Figure 5:**
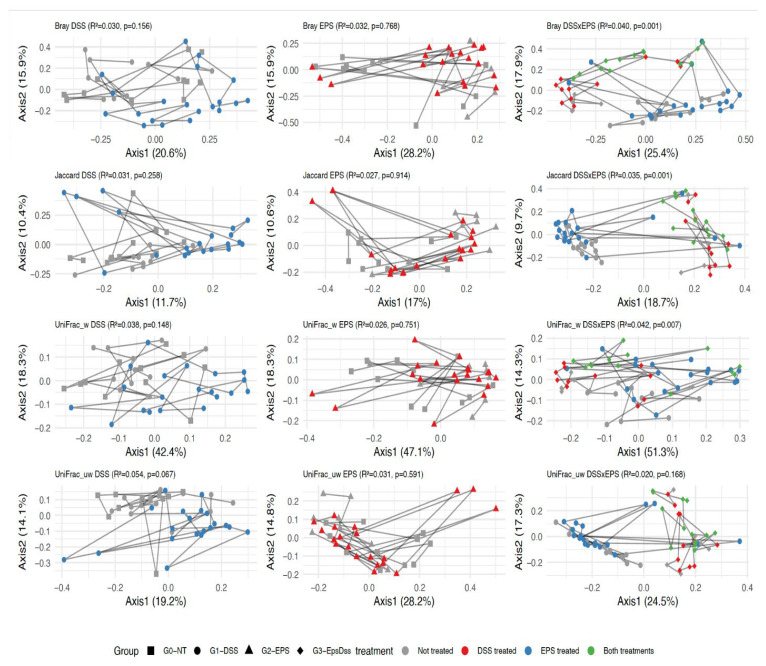
Principal coordinate analysis of beta diversity on WT control mice, as assessed by Bray–Curtis, Jaccard, weighted UniFrac, and unweighted UniFrac distances. Black squares and gray circles indicate the control group; black circles and red circles indicate the groups with DSS-induced colitis; black triangle and blue circle indicate the EPS treated group, and black diamond and green circle indicate the EPS treated group with DSS-induced colitis. Each point represents a single mouse sample, and lines are drawn to connect samples from the same mouse over time.

**Figure 6:**
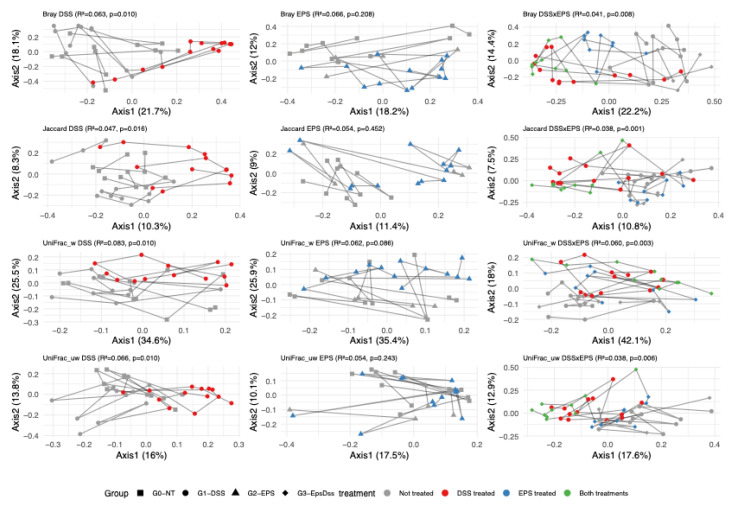
Principal coordinate analysis of beta diversity on Dectin-1 KO mice, as assessed by Bray–Curtis, Jaccard, weighted UniFrac, and unweighted UniFrac distances. Black squares and gray circles indicate the control group; black circles and red circles indicate the groups with DSS-induced colitis; black triangle and blue circle indicate the EPS treated group, and black diamond and green circle indicate the EPS treated group with DSS-induced colitis. Each point represents a single-mouse sample, and lines are drawn to connect samples from the same mouse over time.

To analyze the difference in mice’s microbiota community composition in all experimental conditions, we used PERMANOVA on four distance metrics (Bray–Curtis, Jaccard, weighted UniFrac, and unweighted UniFrac) to inquire whether: (1) the DSS colitis model alters the gut community structure; (2) the EPS alone alters the microbiome structure, and (3) EPS pretreatment would modulate the DSS effect. Figure 5 and Figure 6 display the PCoA plots for the relevant comparisons, along with the R^2^ and *p*-values for the tested treatment effects.

As expected, the gut microbiome differed significantly between genotypes, an effect that was maintained throughout the study period (Supplementary Figures S3 and S4). In WT mice, neither DSS nor EPS alone produced a significant shift in community structure by any metric (Figure 5). The sampling day was a strong covariate across all tests, indicating microbiome drift over time (R^2^ = 4–7%, *p* ≤ 0.001). EPS pretreatment significantly modified the impact of DSS on both Bray–Curtis and Jaccard distances (*p* < 0.05), and unweighted UniFrac showed a modest interaction effect (*p* = 0.015), indicating a potential change in the DSS effect brought about by the EPS pretreatment.

In Dectin-1 KO mice, DSS alone significantly reshaped community composition as measured by Bray–Curtis (*p* ≤ 0.010), Jaccard (*p* ≤ 0.016), and weighted and unweighted UniFrac (*p* ≤ 0.010) (Figure 6). No effects were detected in the gut microbiome structure by EPS treatment alone. However, EPS pretreatment significantly altered the DSS response in KO mice for Bray–Curtis and Jaccard (*p* < 0.02) and unweighted UniFrac (*p* = 0.015). In summary, EPS alone has a minimal impact on the microbiome; however, pretreatment with EPS consistently modifies the DSS-driven community changes in both genotypes, most notably in non-phylogenetic distances.

### 2.5. Alpha Diversity Results

To analyze the richness and evenness of microbial species, we used the alpha diversity, which was not significantly different between genotypes at Day 2 (Baseline) under any of the metrics used. However, a mixed-effect linear model of each α-metric over time (Days 2–11, with a random intercept + slope for each mouse), detected a robust main effect of genotype on Faith’s PD (F = 5.52, *p* = 0.027), with KO mice harboring a higher phylogenetic response than WT mice (Supplementary Figures S5 and S6). Any measure of alpha diversity used detected no significant DSS, EPS, or DSS × EPS treatment effects. Only Faith’s PD exhibited a nominal interaction effect in KO (*p* = 0.012), mirroring the phylogenetic-focused response we saw in β-diversity (Supplementary Figure S6). Dectin-1 deficiency is associated with modest but reproducibly higher phylogenetic α-diversity (Faith’s PD) before and during colitis. At the same time, richness and evenness measures are largely unaffected by genotype or EPS/DSS treatments alone (Supplementary Figure S7).

### 2.6. Differences in Taxonomic Groups

**Figure 7:**
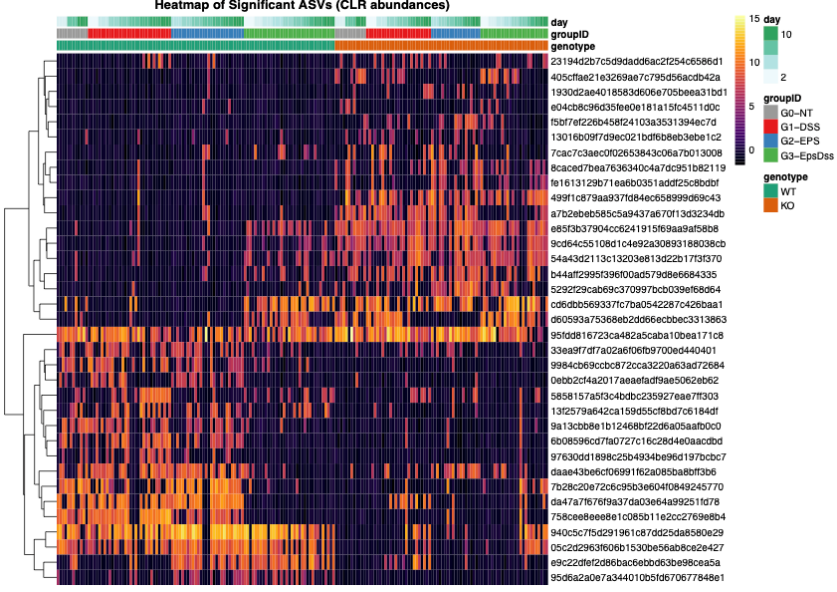
Heatmap showing the ASVs that are differentially abundant between the genotypes, regardless of treatment status. G0—healthy wild-type mice; G1—DSS-induced colitis wild-type mice; G2—EPS-treated wild-type mice, and G3—EPS treated and DSS-induced colitis wild-type mice. The color scale indicates the CLR transformed counts.

To identify the groups of microbial bacteria in the gut, we used a linear mixed-effects model to analyze all untreated samples over time. We detected 35 ASVs whose baseline trajectories differed by genotype, after FDR correction (q < 0.05). Taxonomic annotation of these genotype-associated ASVs revealed that Dectin-1 KO mice had increased amounts of Proteobacteria and sulfate-reducers, namely, *Helicobacter ganmani*, and unknown Deltaproteobacteria, as well as *Mucispirillum schaedleri.* Taxa increased in the WT genotype, including several ASVs classified as *Allobaculum*, *Akkermansia muciniphila*, *Bifidobacterium*, *Ruminococcus bromii*, *Lactobacillus salivarius*, and *Ruminococcus gnavus*, all species associated with a normal microbiome composition (Figure 7).

**Figure 8:**
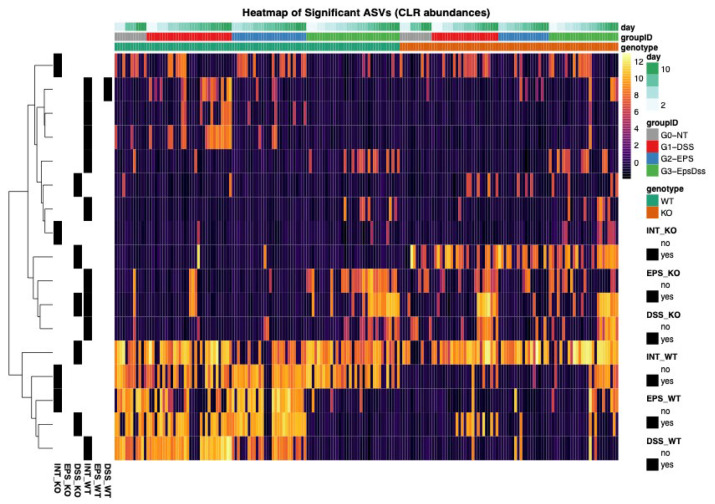
Heatmap showing the ASVs that are differentially abundant for each treatment combination. G0—healthy Dectin-1 KO mice; G1—DSS-induced-colitis Dectin-1 KO mice; G2—EPS-treated Dectin-1 KO mice and G3—EPS treated and DSS-induced-colitis Dectin-1 KO mice. The row annotation indicates in which treatment/genotype combination the ASV was modulated. DSS_WT and DSS_KO—effects of DSS treatment on WT and KO genotypes, EPS_WT and EPS_KO—effects of EPS treatment on WT and KO genotypes, INT_WT and INT_KO—effects of the interaction of DSS and EPS treatments. The color scale indicates the CLR transformed counts.

DSS treatment altered only one ASV in WT mice, an unclassified Alphaproteobacterium (RF32 clade). In Dectin-1 KO mice, DSS provoked significant shifts in *Helicobacter ganmani*, *Paraprevotellaceae* unknown ASV, *Turicibacter*, *Allobaculum*, and unclassified *Clostridiales*. No ASVs responded to EPS treatment by itself in either genotype. However, EPS pretreatment reshaped the DSS-driven shifts in both genotypes. In WT mice, nine ASVs showed significant DSS × EPS interactions, including: an RF32 Alphaproteobacterium, *Bacteroides*, an unclassified S24-7 gut group bacterium, *Odoribacter*, *Paraprevotella*, *Akkermansia muciniphila*, a *Desulfovibrionaceae* family member, and *Parabacteroides*. Four ASVs exhibited significant DSS × EPS interactions in Dectin-1 KO: an unclassified S24-7, *A. muciniphila*, *Bifidobacterium*, and a *Mycoplasmataceae family* ASV. Together, these data show that while DSS triggers only a muted compositional shift in WT, it provokes a broader bloom of pathobionts in Dectin-1 KO mice and that EPS pre-treatment selectively counteracts many of those DSS-induced alterations in both genotypes, albeit acting on a larger and more phylogenetically diverse set of taxa in WT than in KO (Figure 8).

Intestinal homeostasis relies on the composition of the microbiota, the secretion of metabolites (such as short-chain fatty acids, or SCFAs), and the modulation of the mucosal immune response. The colon is known to have a diverse microbial content, supporting the idea that improving colon microbiome abnormalities could be beneficial to individuals suffering from ulcerative colitis and breaking the “one-microbe–one-disease” concept [41,51]. Among the many potential mechanisms of IBD pathogenesis, one involves the decrease in butyrate-producing bacteria, which affects the protection of the intestinal epithelial barrier against potential pathogens, and the increase in members of Bacteroidetes and Proteobacteria, both of which are associated with disease relapse [41,52]. Additionally, some gut bacterial phyla, such as Proteobacteria, promote inflammatory responses due to their Gram-negative-specific lipopolysaccharide (LPS) [48].

Our data showed that DSS-induced colitis treated with EPS, for both genotypes, resulted in the restoration of *Akkermansia muciniphila*, a Gram-negative microorganism essential for maintaining intestinal barrier integrity and overall host health. Its introduction can induce the expression of the intestinal barrier’s tight junction proteins, such as ZO-1 and occludin, reduce the presence of macrophages and cytotoxic T-lymphocytes in the colon, diminishing symptoms of intestinal inflammation, and promote Foxp3+regulatory T cells in the colon, alleviating inflammation and modulating the composition of intestinal microbiota [51,53,54,55]. Other bacteria that improve the intestinal function are *Parabacteroides* and *Bacteroides*, which were increased only in WT DSS-induced colitis treated with EPS. Along with *Alistipes*, they have attracted considerable attention due to their anti-inflammatory properties [56]. *Parabacteroides*, which are considered potentially an anti-inflammatory taxon, are negatively associated with inflammatory bowel diseases (IBD), promote resistance to *Clostridioides difficile* infection, are associated with decreased hepatic steatosis, and exert protective effects on multiple sclerosis, epilepsy, obesity, metabolic dysfunctions, and tumors [56,57,58]. Some *Parabacteroides* spp., such as *Parabacteroides distasonis*, have been extensively studied for their ability to regulate immune responses in both humans and mice, stimulating the expression of both IL-10 and CD4+CD25+ T cells, as well as IL-10+FoxP3+ T regulatory cells [59,60]. *P. distasonis* has also demonstrated its importance in attenuating experimental mouse colitis induced by both dextran sulfate sodium (DSS) and 2,4,6-trinitrobenzenesulfonic acid (TNBS), as well as stabilizing the intestinal microbial ecology in DSS-induced murine colitis [60,61,62]. The EPS treatment and DSS-induced colitis of WT mice increased IL-10 levels, which could be associated with the presence of this bacterium, which was not detected in the DSS-induced colitis group. Another bacterium detected in EPS treatment and DSS-induced colitis of WT mice is *Bacteroides*, which is abundant in mammalian gut microbiota members and may provide colonization resistance to gut pathogens. It has been demonstrated that some *Bacteroides* species have protective effects on animal models of colitis, as shown for *B. uniformis* and *B. vulgatus* (strain Bv46), which can restore the colonic mechanical and immune barriers by inhibiting the secretion of pro-inflammatory cytokines, thus alleviating DSS-induced colitis [56,63]. It was also reported that treatment with bovine milk oligosaccharides supported the growth of *Bifidobacterium longum subsp. longum* and *Pa. distasonis* while diminishing the growth of *Clostridium perfringens* and *Escherichia coli* [60,64], which was partially observed in the EPS treatment and DSS-induced colitis of Dectin-1 KO mice. However, we did not observe an improvement in the colon inflammatory reaction in this group.

Some microbiome members, like S24-7, induce carbohydrate fermentation within the digestive tract. The role of this group of bacteria has been investigated due to its connection with inflammation, particularly in the pathogenesis of IBD, and considerable alterations observed in UC [65]. It was demonstrated that enhancing S24-7 in mice could modulate the MAPK pathway, dampen inflammatory responses by attenuating TNF-α activity, and diminish inflammatory cell infiltrates, along with restructuring the gut microbiota [65,66]. It was also demonstrated that *Ficus carica* heteropolysaccharide attenuated DSS-induced colitis in mice, regulating inflammatory responses and maintaining intestinal endothelial integrity, while elevating the levels of S24-7, *Coprococcus*, *Proteobacteria*, and *Odoribacteraceae*, *as well as Paraprevotellaceae*, in mice, showing great potential for the application of polysaccharides in the prevention or treatment of IBD [65,67].

Our data showed that the EPS treatment and DSS-induced colitis in WT mice resulted in an improvement in the bacteria that produce SCFA, such as *Bacteroides*, *Akkermansia*, *and Parabacteroides*, which was not observed in Dectin-1 KO mice.

## 3. Materials and Methods

### 3.1. Cultivation of Auricularia auricula and Polysaccharide Fraction

The mushroom *Auricularia auricula* (*CC-305*) was obtained from the Germplasm Bank of Mushrooms for Human Use of Embrapa Genetic Resources and Biotechnology (Brasília, Brazil). Access to genetic property was granted by the National Council of Scientific and Technological Development (CNPq), under authorization number no. 010342/2014-1. *Auricularia auricula* were grown, as previously described [33], in Potato Dextrose culture medium (PD) supplemented with 10% agar at 28 °C. Fragments of this mushroom (approximately 5 cm^2^) were inoculated in PD broth supplemented with lactic acid (3.3 mg/mL) and cultivated under rotation (130 rpm) at 30 °C in the dark for 7 days. This culture was expanded by adding culture medium and maintaining growth conditions as above. After 7 days of culture, the mycelia were centrifuged (6000× *g*, 4 °C, 12 min) and washed three times with ultrapure water. Next, mycelia were resuspended in sterile, endotoxin-free water and kept under the conditions mentioned above for 24 h. Finally, the culture was centrifuged, and the supernatant was stored at −20 °C for lyophilization. Lyophilized samples were resuspended in ultrapure water and centrifuged (5000 rpm, 4 °C, 10 min) for fractionation. The supernatant (water-soluble fraction) was collected, and the precipitate containing the insoluble fraction was resuspended with ultrapure water. For the following experiments, we used a suspension of the precipitate fraction, which is identified here as the insoluble fraction containing *Auricularia* polysaccharides. The treatment dose was based on our previously collected data, considering the cytotoxicity and efficacy of EPS [33].

### 3.2. Nuclear Magnetic Resonance (NMR) Spectroscopy

All the NMR measurements were acquired in an 11.7 Tesla (500 MHz for hydrogen frequency), using a temperature of 343 K, as solvent Dimethyl Sulfoxide (DMSO–d4), and the inverse detection probe head except for ^13^C (the broadband direct observe). For the ^1^H spectrum with HDO pre-saturation signal using continuous wave, the parameters were acquisition time (AQ = 3.27 s), sweep width (SWH =10,000 Hz), relaxation delay (d1 = 1 s), the 90° pulse time (p1 = 8.5 µs), and number of scans (ns = 128). For the ^13^C {^1^H} the parameters were acquisition time (AQ = 0.52 s), sweep width (SWH =31,250 Hz), relaxation delay (d1 = 0.1 s), the 90° pulse time (p1 = 9.5 µs), and number of scans (ns = 4 K) for all 2D experiments. For all heteronuclear correlation (HSQC_edit_; HSQC-TOCSY), the SWHF1 10,000 Hz and SWHF2 31,250 Hz, d = 1.5 s, and number of experiments in F1 = 256, AQ in F2 = 0.27 s, and number of scans (ns = 8) were used. The signals of sugar residues were assigned by matching and comparing the chemical shift values presented in the 1D spectrum (1H and 13C spectra) and 2D spectrum (HSQC_edit_ and HSQC-TOCSY spectra). All data were processed using TopSpin software (Bruker TopSkin 4.1.3 Academic License).

### 3.3. Colitis Model Experiment

For the in vivo and ex vivo tests, male *Mus musculus* c57BL/6J and Dectin-1 Knockout mice, aged 8–12 weeks, were used. The number of mice per group was 4, and two independent experiments were conducted. During the experimental period, they were housed in the animal facilities of the Institute of Biological Sciences at the University of Brasília, in ventilated racks with ad libitum access to food and water. The sample size and the use of these animals were stipulated in the project (23106.074021/2021-11), approved by the Ethics Committee on Animal Use of the University of Brasília, following protocols designed to guarantees this study’s statistical integrity while adhering to ethical principles that minimize the number of animals used in the experiment and reduce pain, suffering, and distress. There is no endpoint considering the experiment’s short duration. Before the experimental procedure, the mice were rotated to achieve co-housing stability. The animals were then separated into their respective experimental groups and placed in specific boxes on shelves with micro-isolated ventilation. No exclusion criterion was used to compose the groups or conduct the experiments. To evaluate a tissue inflammatory process, we used the colitis induction model with Dextran Sodium Sulfate (DSS) (Sigma-Aldrich, Aizu, Fukushima Prefecture, Japan) at a concentration of 2%. The experimental grouping of mice and specific treatment occurred as follows:

(G0) Control group (NT): Drinking water was given to mice throughout the experimental period. Then, the same volume of phosphate-buffered saline (PBS) was administered by gavage during the experiment;

(G1) DSS 2% group (DSS): The mice were given drinking water containing 2% DSS from Day 6 to 10 of the experimental period;

(G2) AaIns. Polysaccharide group (EPS): Drinking water was given to mice throughout the experimental period. Then, 100 µL/day of a 1 mg/mL suspension of AaIns polysaccharide dissolved in water was administered by gavage during the experiment for days 6–10 of the intervention;

(G3) DSS 2% + AaIns. Polysaccharide group (EPSxDSS): Mice were given drinking water containing 2% DSS for Days 6–10 of the intervention. Then, 100 µL/day of a 1 mg/mL suspension of AaIns polysaccharide dissolved in water was administered by gavage during the experiment, from Days 3 to 10 of intervention.

During the experimental period, feces were collected from all mice in the groups and stored at −80 °C. The fecal contents were collected in sterile containers for DNA sequencing analysis as mentioned in 3.6. On the eleventh day, all the animals were anesthetized for blood collection and then euthanized. The mice’s intestines were removed to assess the length of the cecum to the rectum. Photographs were taken with a measurement standard for comparative analysis, and bowel length was analyzed using ImageJ 1.53t software. After that, the mice’s intestines were sectioned for cytokines and histopathological study.

### 3.4. Cytokine Quantification

Cytokine quantification was performed using the ELISA (Enzyme-Linked Immunosorbent Assay) technique, using specific Invitrogen brand kits, following the manufacturer’s instructions. As inflammatory profile markers of disease evolution, we quantified TNF-α, IL-10, IL-13, IFN-γ, IL-17A, and IL-23 (Invitrogen, Vienna, Austria) in the intestinal tissue lysate (colonic portion) of mice.

### 3.5. Histopathological Analysis

The collected biological material (colon) was placed in previously labeled cassettes and stored in a buffered formaldehyde solution (10%) until sample processing. The tissues were processed and placed in paraffin blocks, which were cut to prepare the histological section slides. The slides for histopathological analysis were stained with Hematoxylin–Eosin (HE) and scanned using the Aperio Scanner CS2 equipment (Leica Biosystems, Nussloch, Germany). The acquired images were analyzed using the Aperio ImageScope x64 software (version 12.4.6.5003) and analyzed in a blinded manner. A blinded observer conducted the histopathological analysis.

### 3.6. 16S rRNA Gene Sequencing and Taxonomic Assignment

Genomic DNA was extracted from stool using the Qiagen DNA PowerSoil Pro Kit, which included an initial bead-beating step with the environmental lysing matrix E. The V4 region of 16S rRNA was then amplified and sequenced on an Illumina MiSeq in a blinded manner. Reads were processed in QIIME 2 2023.2 using DADA2 for denoising and ASV calling. Taxonomy was assigned against the SILVA 138 database.

### 3.7. Diversity Analysis

An ASV table was generated and rarefied to a uniform depth (1138 sequences) for α-diversity analysis. Four metrics, namely the ASVs, Shannon diversity, Pielou’s evenness, and Faith’s phylogenetic diversity, were computed with QIIME 2. Four β-diversity distance matrices were calculated: Bray–Curtis, Jaccard, and weighted and unweighted UniFrac.

### 3.8. α-Diversity Statistics

At baseline (Day 2), metric differences by genotype were tested using the Wilcoxon rank-sum test. Longitudinal α-diversity (Days 2–11) was modeled in R (v.4.4.2) using linear mixed-effects models (packages lme4, lmerTest). An initial test was conducted with all untreated samples to determine genotype differences. Treatments were evaluated using 3 separate models to evaluate whether (1) the DSS colitis model alters the gut community structure (group NT × group DSS), (2) the EPS alone alters the microbiome structure (group NT × group EPS), or (3) EPS pretreatment would modulate the DSS effect (group DSS × group EPSxDSS). A random intercept (each mouse) and slope (day) were used in these models. Type III ANOVA (Satterthwaite) assessed main and interaction effects.

### 3.9. β-Diversity Statistics

The same strategy used for alpha diversity was applied to test effects on β-Diversity: an initial analysis of differences in genotypes, followed by treatment tests as described. For these tests, we used PERMANOVA (package vegan, function adonis2, 999 permutations, by = “margin”). Matrices were filtered to contain the appropriate samples, to isolate the effects, and allow for marginal tests. Sampling day was used as a fixed effect in all tests, and within-group mouse IDs as a blocking factor (strata). Results were visualized using principal coordinate analysis.

### 3.10. ASV-Level Modeling

ASV counts were transformedthi using centered log-ratio (CLR) transformation performed via ALDEx2 (128 Monte Carlo instances), using the unrarefied counts and all samples with at least 400 sequences. For each ASV, CLR values across all non-treated samples (Days 2–11) were fit to a mixed model as

$$clr_{ij}=\beta^{0}+\beta^{1}\cdot Genotype_{i}+\beta^{2}\cdot Day_{j}+b_{0i}+b_{1i}\cdot Day_{j}+\varepsilon_{ij},$$

with

$$\left(b_{0i}b_{1i}\;\right)∼N!\left(\left(00\;\right),\left(\sigma_{b0}^{2}\;\sigma_{b0b1}\;\sigma_{b0b1}\;\sigma_{b1}^{2}\;\right)\right),\;\varepsilon_{ij}∼N\left(0,\sigma^{2}\right)$$

where

*clr*_ij_: CLR-transformed abundance of a given ASV in sample j from mouse I;

β^0^: fixed intercept;

β^1^: fixed effect of *Genotype_i_*, coded as 1 for KO vs. 0 for WT;

β^2^: fixed effect of Dayj_jj (sampling days 2–11);

*b*_0*i*_: random intercept for subject (mouse) *I*;

*b*_1*i*_: random slope on Day for subject *i*.

$\left(b_{0i}b_{1i}\;\right)∼N!\left(\left(00\;\right),\left(\sigma_{b0}^{2}\;\sigma_{b0b1}\;\sigma_{b0b1}\;\sigma_{b1}^{2}\;\right)\right)$: random effects are bivariate normal with variances $\sigma_{b0}^{2}\sigma_{b1}^{2}$ and covariance $\sigma_{b0b1}$

$\varepsilon_{ij}∼N\left(0,\sigma^{2}\right)$: residual error

Transformed counts were tested only if the ASV was present in all mice within a group at any given time point. Type III ANOVA *p*-values were Benjamini–Hochberg corrected (q < 0.05) to identify genotype-associated taxa. Similar models, including DSS, EPS, and DSS × EPS terms, were run per genotype to determine treatment and interaction responders.

### 3.11. Statistical Analysis

Results of both biological replicates of the experiment were expressed as mean ± SD of experimental data and analyzed using GraphPad Prism (version 8.0.1). For the colon length analysis, we applied two-way analysis of variance (ANOVA), followed by Sidak’s multiple comparisons test. For cytokine quantification, one-way analysis of variance (ANOVA) was used, followed by Tukey’s test to account for multiple comparisons. *p*-values less than or equal to 0.05 were considered statistically significant, and higher *p*-values were considered not significant (NS).

## 4. Conclusions

Our data support the proposal that carbohydrate-based compounds have therapeutic potential in treating inflammatory intestinal diseases. We demonstrated that this is possible through a combination of various factors, including the increase in SCFA-producing microbiota bacteria and the production of metabolites that stimulate the secretion of gut homeostatic cytokines, such as IL-10 and TNF-α, which are dependent on Dectin-1 receptor expression. Additional studies are needed before these data can be extrapolated to human colitis patients.

## Data Availability

The original contributions presented in this study are included in the article/Supplementary Material. Further inquiries can be directed to the corresponding author.

## Supplementary Materials

The following supporting information can be downloaded at https://www.mdpi.com/article/10.3390/ph18081085/s1, Table S1. ^13^C and ^1^H assignments of the polysaccharides from *Auricularia auricula*, Table S2. ASV from treatments; Table S3. Differences between wild-type and Dectin-1 KO mice. Figure S1—Morphological evaluation of DSS-induced colitis in wild-type mice colon; Figure S2—Morphological evaluation of DSS-induced colitis in Dectin-1 KO mice; Figure S3. Beta diversity differences observed as principal coordinate analysis (PCoA); Figure S4. Beta diversity analysis; Figure S5. Alpha diversity between genotypes; Figure S6. Alpha Diversity analysis over time by treatment and genotype; Figure S7. Alpha Diversity observed over time in all groups.
