# Supplementary material for: Auricularia auricula’s Exopolysaccharide Mitigates DSS-Induced Colitis Through Dectin–1-Mediated Immunomodulation and Microbiota Remodeling
Source: Pharmaceuticals (Basel). 2025 Jul 22;18(8):1085. doi: 10.3390/ph18081085 (PMC12389670; doi:10.3390/ph18081085)
Supplement: Supplementary file 1 [file pharmaceuticals-18-01085-s001.zip › pharmaceuticals-3707808-supplementary.pdf]

Article

Auricularia auricula's Exopolysaccharide Mitigates DSS-Induced Colitis through Dectin–1–Mediated Immunomodulation and Microbiota Remodeling

Luísa Coutinho Coelho[1, #], Luísa Dan Favilla [1, #], Thaís Bergmann de Castro[1], Maria Carolina B. Di Medeiros Leal [2], Christian Hoffmann [3], and Anamélia Lorenzetti Bocca [1,4, *]

Supplementary Tables and Figures

Supplementary Table S1. ^{13}C and ^{1}H assignments of the polysaccharides from *Auricularia auricula*

**Units**	**1**	**2**	**3**	**4**	**5**	**6**
**α-Glu*p*-**	**96.8**	*72.2*	73.5	70.5	71.7	*62.0*
	**4.28**	*3.38*	3.63	3.83	3.16	*3.44*
**β -Glu*p*-**	**92.2**	74.4	76.7	71.0	76.7	62.0
	**4.92**	*3.39*	3.16	3.62	3.12	3.33
**-α-Fuc*p*-**	**99.5**	71.11	72.6	76.2	70.0	18.27
	**5.12**	*3.57*	3.68	3.07	4.11	1.15
**β-Man*p*-**	**101.5**	72.9	76.2	69.0	78.5	63.5
	**5.60**	4.04	3.07	3.59	3.39	3.55
**α-Gal*p*-**	**99.4**	84.3	72.6	73.0	71.1	70.6
	**5.44**	3.85	4.04	3.68	3.57	3.83

Supplementary Table S2. ASV from treatments.

**ASV**	**kingdom**	**phylum**	**class**	**order**	**family**	**genus**	**species**
df0e3d38eec730326754d8c17a8b8efe	k__Bacteria;	p__Firmicutes;	c__Bacilli;	o__Turicibacterales;	f__Turicibacteraceae;	g__Turicibacter;	s__
da47a7f676f9a37da03e64a99251fd78	k__Bacteria;	p__Firmicutes;	c__Erysipelotrichi;	o__Erysipelotrichales;	f__Erysipelotrichaceae;	g__Allobaculum;	s__
7b28c20e72c6c95b3e604f0849245770	k__Bacteria;	p__Verrucomicrobia;	c__Verrucomicrobiae;	o__Verrucomicrobiales;	f__Verrucomicrobiaceae;	g__Akkermansia;	s__muciniphila
758cee8eee8e1c085b11e2cc2769e8b4	k__Bacteria;	p__Bacteroidetes;	c__Bacteroidia;	o__Bacteroidales;	f__Bacteroidaceae;	g__Bacteroides;	s__
05c2d2963f606b1530be56ab8ce2e427	k__Bacteria;	p__Actinobacteria;	c__Actinobacteria;	o__Bifidobacteriales;	f__Bifidobacteriaceae;	g__Bifidobacterium	
9091e21c25fbf76afd1d52aeb7fb8823	k__Bacteria;	p__Bacteroidetes;	c__Bacteroidia;	o__Bacteroidales;	f__[Paraprevotellaceae];	g__Paraprevotella;	s__
499f1c879aa937fd84ec658999d69c43	k__Bacteria;	p__Proteobacteria;	c__Epsilonproteobacteria;	o__Campylobacterales;	f__Helicobacteraceae;	g__Helicobacter;	s__ganmani
634e293920d238dfa24961d096247760	k__Bacteria;	p__Bacteroidetes;	c__Bacteroidia;	o__Bacteroidales;	f__Porphyromonadaceae;	g__Parabacteroides;	s__
7d02f99e2ea00512b055a4e70f84c18b	k__Bacteria;	p__Bacteroidetes;	c__Bacteroidia;	o__Bacteroidales;	f__[Odoribacteraceae];	g__Odoribacter;	s__

72e530a4206f2e4804d502f4dfca8387	k__Bacteria;	p__Bacteroidetes;	c__Bacteroidia;	o__Bacteroidales;	f__S24-7;	g__;	s__
9faa90542b330fa9e0c926e24267ec99	k__Bacteria;	p__Proteobacteria;	c__Alphaproteobacteria;	o__RF32;	f__;	g__;	s__
54eaec788f0a38ac8cc0932220c7e94d	k__Bacteria;	p__Bacteroidetes;	c__Bacteroidia;	o__Bacteroidales;	f__S24-7;	g__;	s__
2eb2fe0471e37d54b5e1c47660220ad6	k__Bacteria;	p__Bacteroidetes;	c__Bacteroidia;	o__Bacteroidales;	f__S24-7;	g__;	s__
fe0cb15293a1a84be1bf61a2bdb65a4c	k__Bacteria;	p__Proteobacteria;	c__Deltaproteobacteria;	o__Desulfovibrionales;	f__Desulfovibrionaceae;	g__;	s__
a6b8bcc8499b4128678a3a671a0bd004	k__Bacteria;	p__Firmicutes;	c__Clostridia;	o__Clostridiales;	f__;	g__;	s__
69a11f927915e2a3ba1f7b9c84486527	k__Bacteria;	p__Proteobacteria;	c__Betaproteobacteria;	o__Burkholderiales;	f__Alcaligenaceae;	g__Sutterella;	s__
17cddb82ce2f004cd99f8fb8e90daae2	k__Bacteria;	p__Tenericutes;	c__Mollicutes;	o__Mycoplasmatales;	f__Mycoplasmataceae;	g__;	s__

Supplementary Table S3. Difference between Wild-type and Dectin-1 Knock-out mice.

ASV	mean_clr_KO	mean_clr_WT	sd_clr_KO	sd_clr_WT	n_KO	n_WT	phylum	class	order	family	genus	species
05c2d2963f606b1530be56ab8ce2e427	-0.29775073	7.19259122	1.1253697	3.9111504	36	39	p__Actinobacteria;	c__Actinobacteria;	o__Bifidobacteriales;	f__Bifidobacteriaceae;	g__Bifidobacterium	
0ebb2cf4a2017aeaefadf9ae5062eb62	-0.26475729	2.88483106	1.5473809	4.0319215	36	39	p__Bacteroidetes;	c__Bacteroidia;	o__Bacteroidales;	f__S24-7;	g__;	s__
13016b09f7d9ec021bdf6b8eb3ebe1c2	1.55623363	-0.1966668	3.0453439	1.2275734	36	39	p__Firmicutes;	c__Clostridia;	o__Clostridiales;	f__Ruminococcaceae		
13f2579a642ca159d55cf8bd7c6184df	-0.07132313	2.03249589	1.8159712	3.9426437	36	39	p__Firmicutes;	c__Clostridia;	o__Clostridiales;	f__Lachnospiraceae		
1930d2ae4018583d606e705beeea31bd1	0.56497093	-0.54025557	2.7160666	0.2796855	36	39	p__Bacteroidetes;	c__Bacteroidia;	o__Bacteroidales;	f__S24-7;	g__;	s__
23194d2b7c5d9dadd6ac2f254c6586d1	1.13544258	-0.4342924	3.1032981	0.307556	36	39	p__Firmicutes;	c__Clostridia;	o__Clostridiales;	f__Lachnospiraceae;	g__[Ruminococcus];	s__gnavus
33ea9f7df7a02a6f06fb9700ed440401	0.40536244	3.9824602	2.3775875	4.1774502	36	39	p__Bacteroidetes;	c__Bacteroidia;	o__Bacteroidales;	f__S24-7;	g__;	s__
405cffae21e3269ae7c795d56acdb42a	3.09762434	-0.43733369	4.3538596	0.3495437	36	39	p__Bacteroidetes;	c__Bacteroidia;	o__Bacteroidales;	f__Bacteroidaceae;	g__Bacteroides;	s__
499f1c879aa937fd84ec658999d69c43	4.82106665	-0.36006718	4.6846208	0.3211533	36	39	p__Proteobacteria;	c__Epsilonproteobacteria;	o__Campylobacterales;	f__Helicobacteraceae;	g__Helicobacter;	s__ganmani
5292f29cab69c370997bcb039ef68d64	2.68472534	-0.26469903	3.8077568	1.0057238	36	39	p__Bacteroidetes;	c__Bacteroidia;	o__Bacteroidales;	f__Bacteroidaceae;	g__Bacteroides	
54a43d2113c13203e813d22b17f3f370	3.09054282	0.42638507	3.7624944	2.2767601	36	39	p__Bacteroidetes;	c__Bacteroidia;	o__Bacteroidales;	f__[Odoribacteraceae];	g__Odoribacter;	s__
5858157a5f3c4bdbc235927eae7ff303	-0.47400282	0.90429496	0.3245362	2.8020654	36	39	p__Bacteroidetes;	c__Bacteroidia;	o__Bacteroidales;	f__S24-7;	g__;	s__
6b08596cd7fa0727c16c28d4e0aacdbd	-0.50156808	2.50966759	0.3647812	4.2472663	36	39	p__Firmicutes;	c__Clostridia;	o__Clostridiales;	f__Ruminococcaceae;	g__Ruminococcus;	s__callidus
758cee8eee8e1c085b11e2cc2769e8b4	-0.41951341	5.83931737	0.3430049	4.521842	36	39	p__Bacteroidetes;	c__Bacteroidia;	o__Bacteroidales;	f__Bacteroidaceae;	g__Bacteroides;	s__
7b28c20e72c6c95b3e604f0849245770	-0.27432682	5.67316661	1.1932117	5.1119107	36	39	p__Verrucomicrobia;	c__Verrucomicrobiae;	o__Verrucomicrobiales;	f__Verrucomicrobiaceae;	g__Akkermansia;	s__muciniphila

7cac7c3aec0f02653843c06a7b013008	2.24097759	-0.12071221	4.5501042	1.6629543	36	39	p__Deferribacteres;	c__Deferribacteres;	o__Deferribacterales;	f__Deferribacteraceae;	g__Mucispirillum;	s__schaedleri
8caced7bea7636340c4a7dc951b82119	3.08533794	-0.43068601	4.1694142	0.3212452	36	39	p__Firmicutes;	c__Clostridia;	o__Clostridiales;	f__;	g__;	s__
940c5c7f5d291961c87dd25da8580e29	-0.45950203	9.07821631	0.3196642	4.5702358	36	39	p__Firmicutes;	c__Erysipelotrichi;	o__Erysipelotrichales;	f__Erysipelotrichaceae;	g__Allobaculum;	s__
95d6a2a0e7a344010b5fd670677848e1	-0.54065823	1.51662209	0.3507302	3.2153664	36	39	p__Actinobacteria;	c__Coriobacteriia;	o__Coriobacteriales;	f__Coriobacteriaceae;	g__;	s__
95fdd816723ca482a5caba10bea171c8	9.84153309	6.54726745	3.8043354	4.5807636	36	39	p__Firmicutes;	c__Bacilli;	o__Lactobacillales;	f__Lactobacillaceae;	g__Lactobacillus;	s__salivarius
97630dd1898c25b4934be96d197bcbc7	-0.49575955	1.14408447	0.2961583	3.1676538	36	39	p__Firmicutes;	c__Clostridia;	o__Clostridiales;	f__Lachnospiraceae;	g__Coprococcus;	s__
9984cb69ccbc872cca3220a63ad72684	-0.44945715	3.72603855	0.3101	4.3413125	36	39	p__Firmicutes;	c__Clostridia;	o__Clostridiales;	f__Ruminococcaceae;	g__Ruminococcus;	s__bromii
9a13cbb8e1b12468bf22d6a05aafb0c0	-0.49090926	3.90532956	0.351424	4.2018821	36	39	p__Bacteroidetes;	c__Bacteroidia;	o__Bacteroidales;	f__Rikenellaceae;	g__;	s__
9cd64c55108d1c4e92a30893188038cb	4.95599454	-0.05511389	3.2724919	1.5066497	36	39	p__Bacteroidetes;	c__Bacteroidia;	o__Bacteroidales;	f__;	g__;	s__
a7b2ebeb585c5a9437a670f13d3234db	4.96753804	-0.48213796	4.2131326	0.3184418	36	39	p__Proteobacteria;	c__Deltaproteobacteria;	o__Desulfovibrionales;	f__Desulfovibrionaceae;	g__;	s__
b44aff2995f396f00ad579d8e6684335	3.80499167	0.13952651	4.3581121	2.0729717	36	39	p__Bacteroidetes;	c__Bacteroidia;	o__Bacteroidales;	f__S24-7;	g__;	s__
cd6dbb569337fc7ba0542287c426baa1	5.09247615	1.38767356	5.2062131	3.8809226	36	39	p__Firmicutes;	c__Erysipelotrichi;	o__Erysipelotrichales;	f__Erysipelotrichaceae;	g__Allobaculum;	s__
d60593a75368eb2dd66ecbbec3313863	5.66177071	0.82188868	4.974423	2.9532785	36	39	p__Bacteroidetes;	c__Bacteroidia;	o__Bacteroidales;	f__S24-7;	g__;	s__
da47a7f676f9a37da03e64a99251fd78	0.06181418	5.58854009	2.3508852	5.1539814	36	39	p__Firmicutes;	c__Erysipelotrichi;	o__Erysipelotrichales;	f__Erysipelotrichaceae;	g__Allobaculum;	s__
daae43be6cf06991f62a085ba8bff3b6	1.36983161	5.1083866	3.7036361	4.3420344	36	39	p__Bacteroidetes;	c__Bacteroidia;	o__Bacteroidales;	f__S24-7;	g__;	s__
e04cb8c96d35fee0e181a15fc4511d0c	0.75890102	-0.38669786	3.0187128	0.2882705	36	39	p__Firmicutes;	c__Clostridia;	o__Clostridiales;	f__Lachnospiraceae;	g__;	s__
e85f3b37904cc6241915f69aa9af58b8	6.85682339	0.35635566	2.6220561	2.3533015	36	39	p__Bacteroidetes;	c__Bacteroidia;	o__Bacteroidales;	f__;	g__;	s__
e9c22dfef2d86bac6ebbd63be98cea5a	0.13571211	3.46351977	2.0769517	4.5836838	36	39	p__Firmicutes;	c__Erysipelotrichi;	o__Erysipelotrichales;	f__Erysipelotrichaceae;	g__Allobaculum;	s__
f5bf7ef226b458f24103a3531394ec7d	1.25516595	-0.44710172	3.0798562	0.3191058	36	39	p__Bacteroidetes;	c__Bacteroidia;	o__Bacteroidales;	f__Rikenellaceae;	g__;	s__
fe1613129b71ea6b0351addf25c8bdbf	2.2220543	-0.37520922	4.0407509	0.3310785	36	39	p__Firmicutes;	c__Clostridia;	o__Clostridiales;	f__Ruminococcaceae;	g__Ruminococcus;	s__

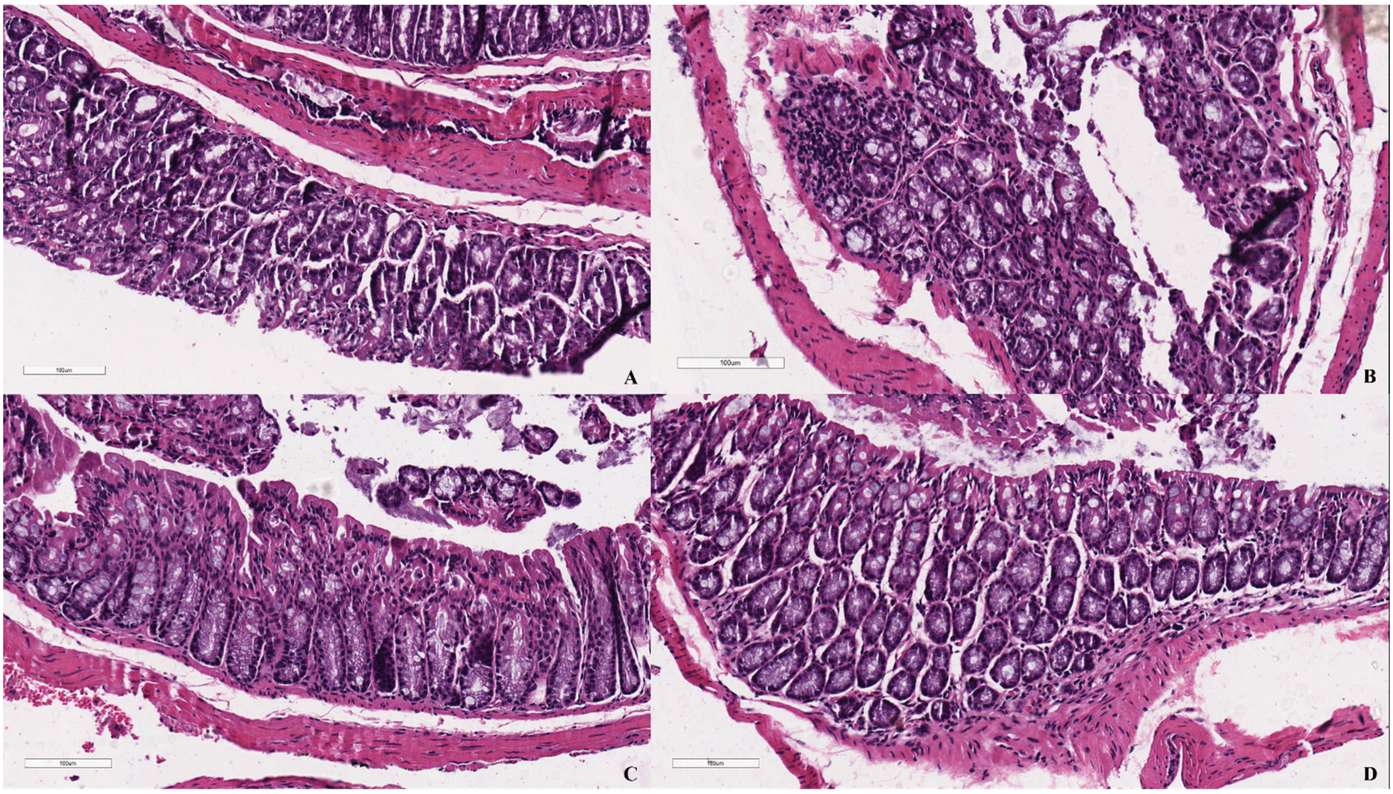

Supplementary Figure S1. Morphological evaluation of DSS-induced-colitis in wild-type mice colon. (A) control mice, (B) DSS-induced-colitis group, (C) EPS treatment group, (D) EPS-treatment and DSS-induced-colitis group. HE, magnification 200×; Results represent two independent experiments, each with five animals per experimental group/

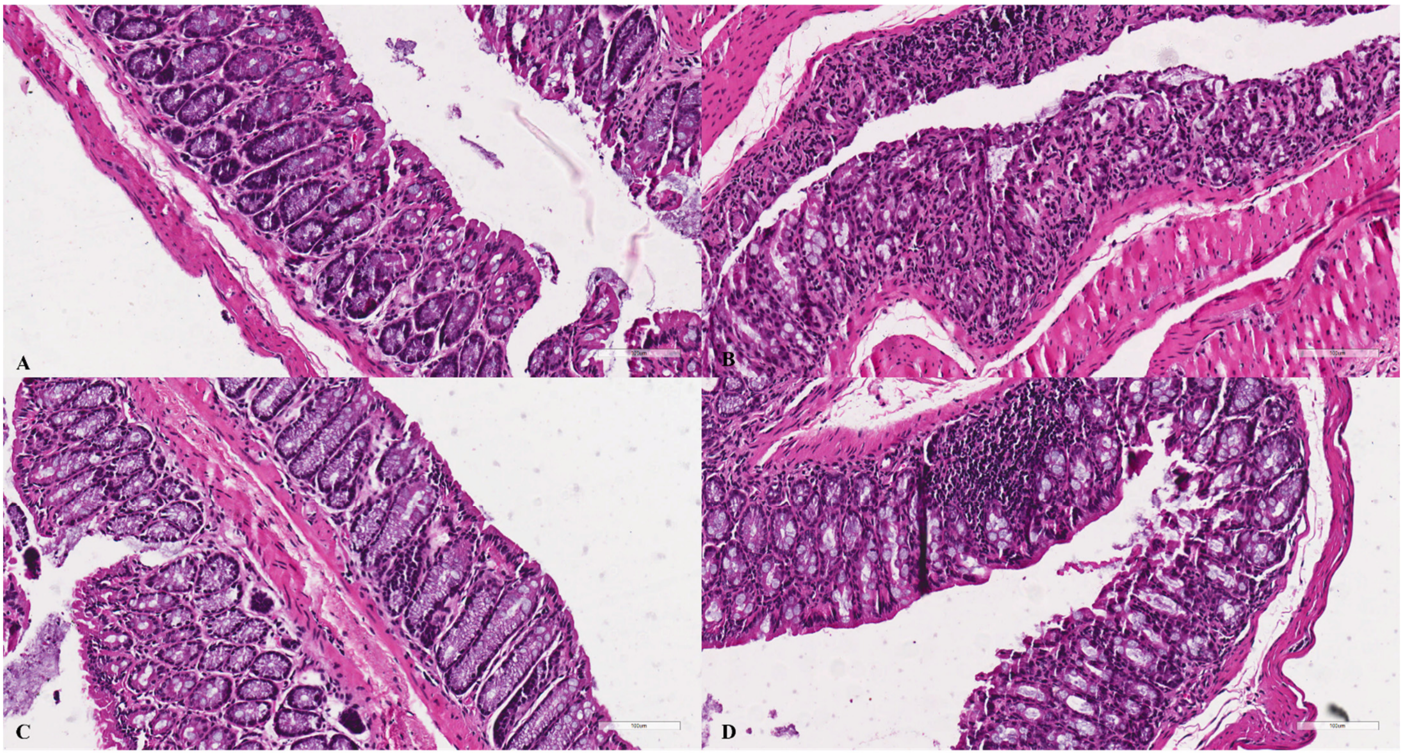

Supplementary Figure S2. Morphological evaluation of DSS-induced-colitis in Dectin-1 KO mice colon. (A) Control mice, (B) DSS-induced-colitis group, (C) EPS treatment group, (D) EPS-treatment and DSS-induced-colitis group. HE, magnification 200×; Results represent two independent experiments, each with five animals per experimental group.

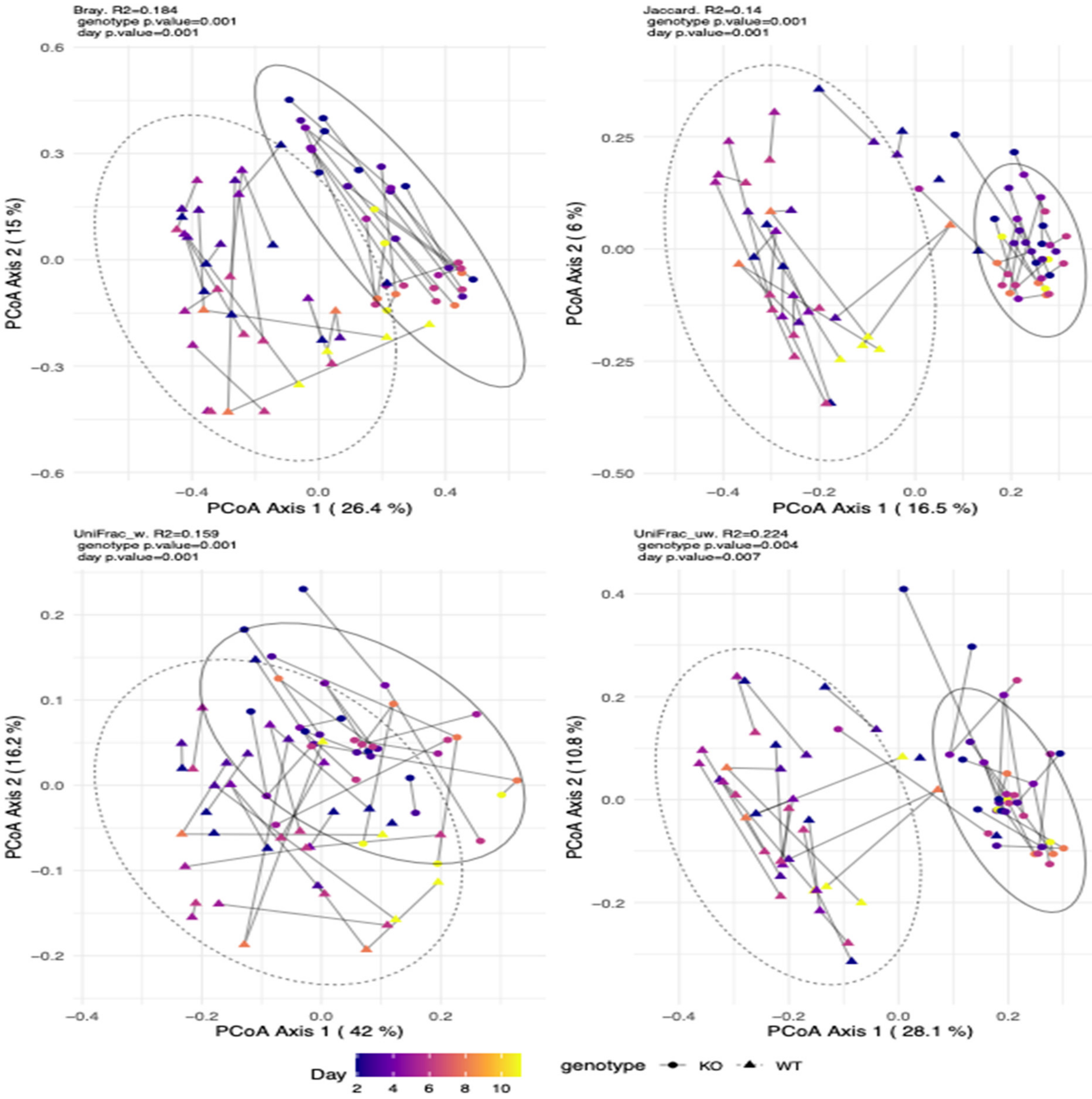

Supplementary Figure S3. Beta diversity differences observed as Principal Coordinate Analysis (PCoA) at day 2, and over time for all non-treated samples, comparing KO- Dectin-1 Knock-out mice from all groups and WT- Wild-type mice from all groups (PERMANOVA p values shown – adonis2 test).

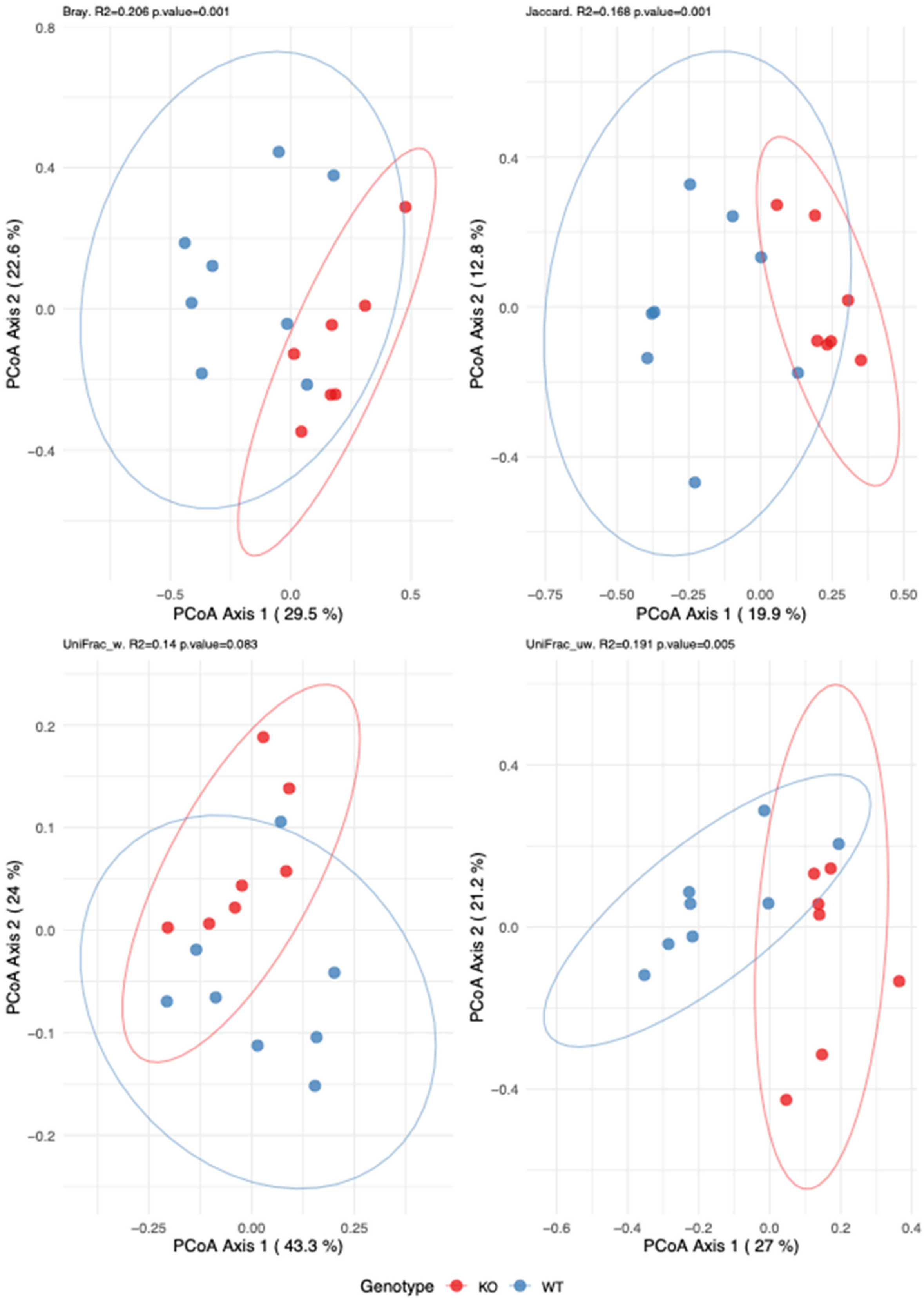

Supplementary Figure S4. Beta diversity analysis. Differences observed on day 2 depicted KO- Dectin-1 Knock-out mice from all groups and WT- Wild-type mice from all groups over time for all non-treated samples.

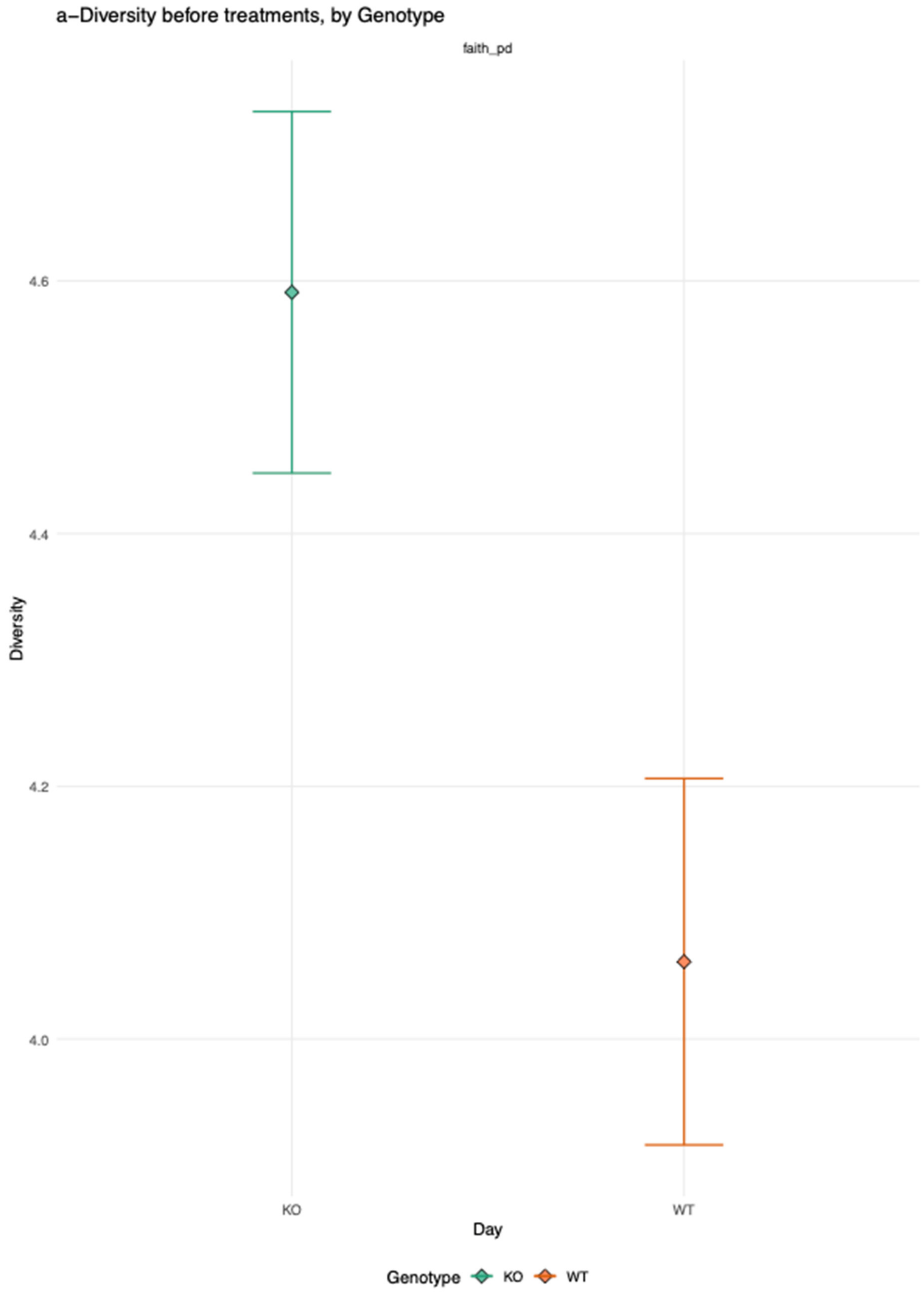

Supplementary Figure S5. Alpha diversity between genotypes was analyzed at day 2 before treatment with EPS. KO- Dectin-1 Knock-out mice from all groups and WT- Wild-type mice from all groups before treatment. It was observed that there was a statistically significant difference between genotypes.

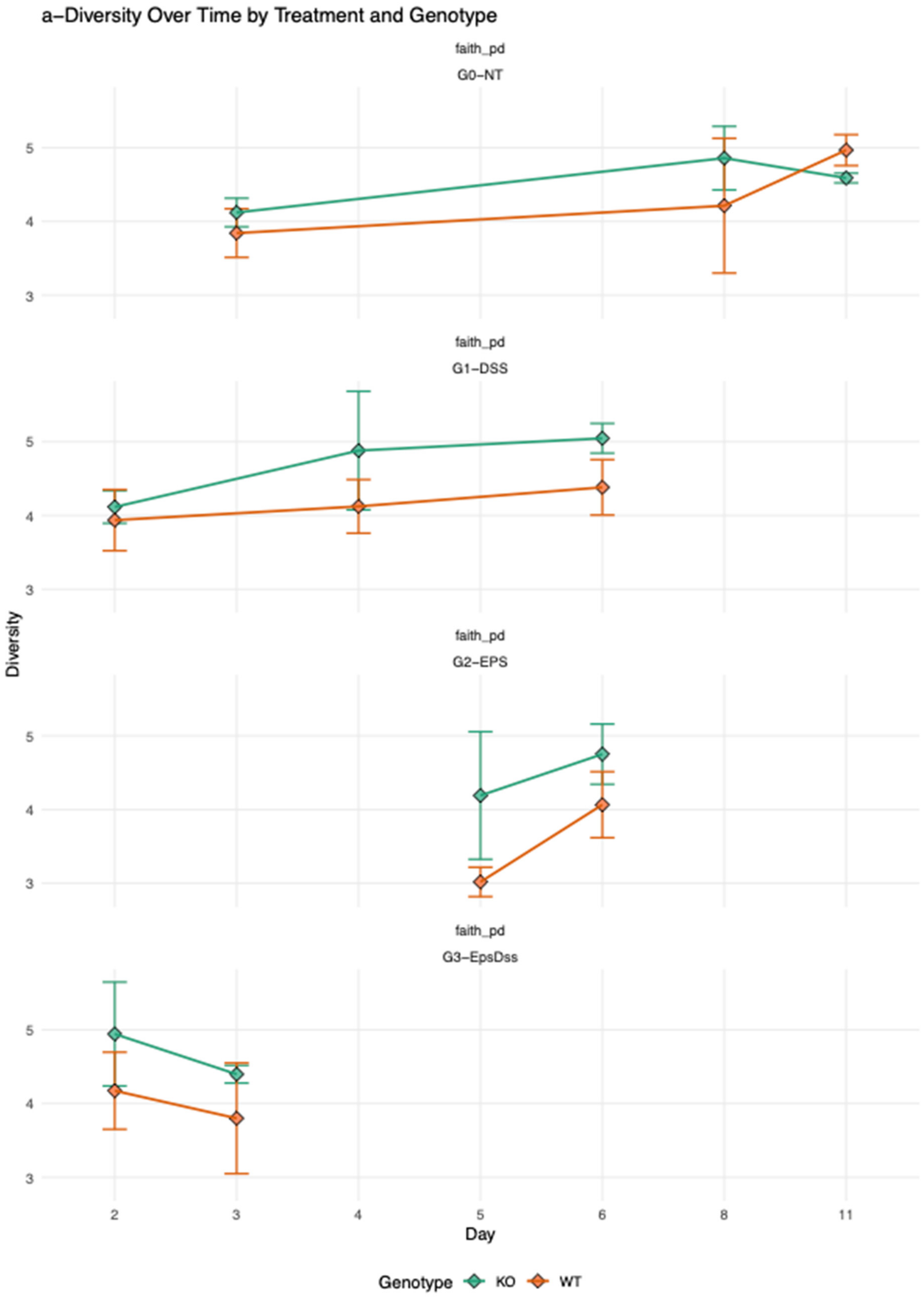

Supplementary Figure S6. Alpha Diversity analysis over time by treatment and genotype, where KO- Dectin-1 Knock-out mice from all groups and WT- Wild-type mice from all groups. Faith's phylogenetic diversity is smaller in the WT genotype (linear mixed effect model, F = 5.52, p <= 0.027).

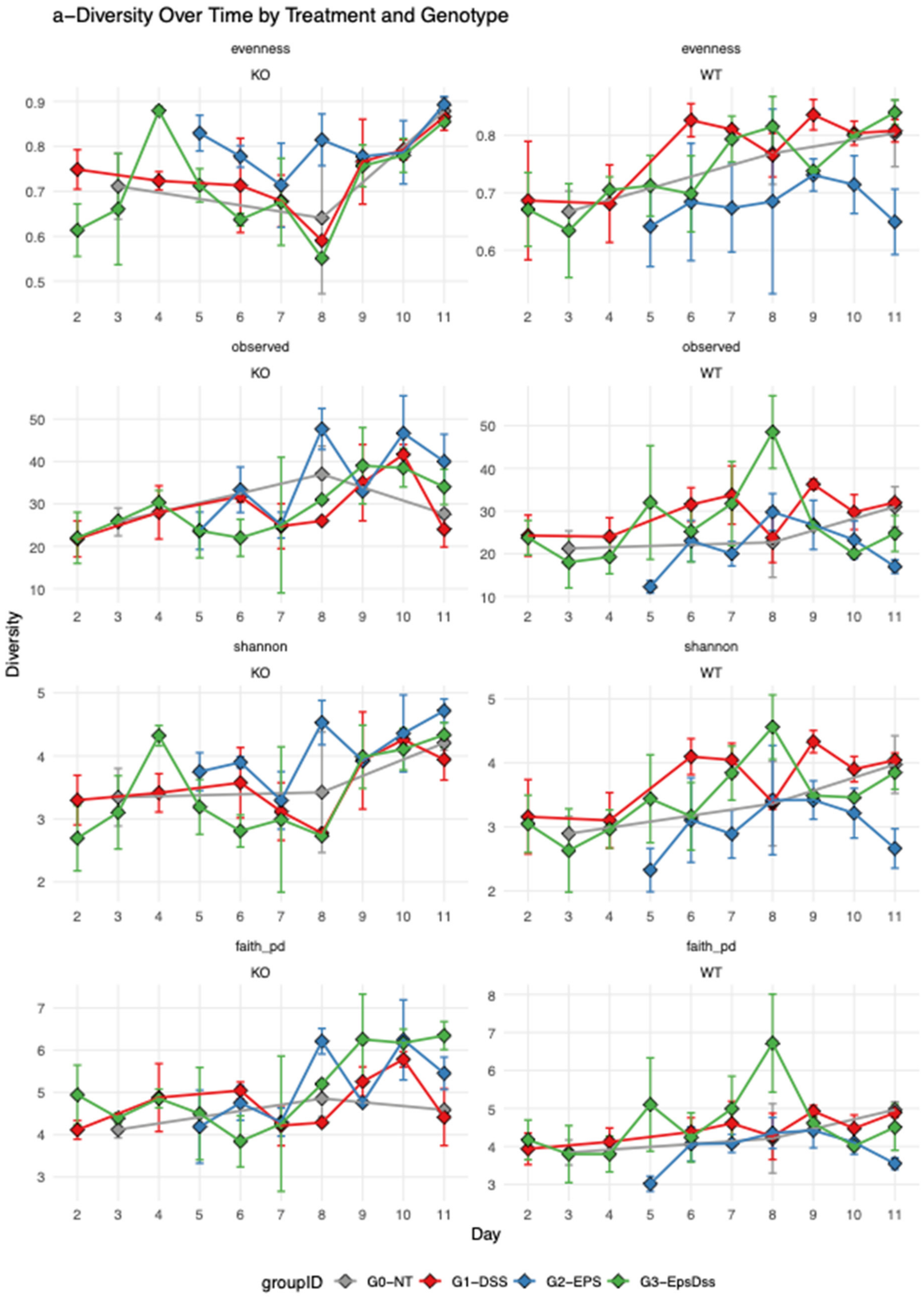

Supplementary Figure S7. Alpha Diversity observed over time in all groups where KO- Dectin-1 Knock-out mice and WT- Wild-type mice. G0 - healthy mice; G1 - DSS-induced colitis mice; G2 - EPS-treated mice, and G3 - EPS treated and DSS-induced colitis mice. Subtle effects of treatments could be discerned in alpha diversity, but effects between genotype and temporal effects were much higher.
